# Effectiveness of intensive group and individual interventions for smoking cessation in primary health care settings: a randomized trial

**DOI:** 10.1186/1471-2458-10-89

**Published:** 2010-02-23

**Authors:** Maria Ramos, Joana Ripoll, Teresa Estrades, Isabel Socias, Antonia Fe, Rosa Duro, Maria José González, Margarita Servera

**Affiliations:** 1 Deparment of Public Health, Balearic Department of Health, Palma, Spain; 2 Primary Health Care Mallorca District, Balearic Health Service, Palma, Spain

## Abstract

**Objectives:**

Primary: To compare the effectiveness of intensive group and individual interventions for smoking cessation in a primary health care setting; secondary: to identify the variables associated with smoking cessation.

**Methods:**

Three-pronged clinical trial with randomisation at the individual level. We performed the following: an intensive individual intervention (III), an intensive group intervention (IGI) and a minimal intervention (MI). Included in the study were smokers who were prepared to quit smoking. Excluded from the study were individuals aged less than 18 years or with severe mental conditions or terminal illnesses. The outcome measure was continued abstinence at 12 months confirmed through CO-oximetry (CO). The analysis was based on intention to treat.

**Results:**

In total, 287 smokers were recruited: 81 in the III, 111 in the IGI, and 95 in the MI. Continued abstinence at 12 months confirmed through CO was 7.4% in the III, 5.4% in the IGI, and 1% in the MI. No significant differences were noted between III and MI on the one hand, and between IGI and MI on the other [RR 7.04 (0.9-7.2) and RR 5.1 (0.6-41.9), respectively]. No differences were noted between IGI and III [RR 0.7 (0.2-2.2)]. In multivariate analysis, only overall visit length showed a statistically significant association with smoking cessation.

**Conclusions:**

The effectiveness of intensive smoking interventions in this study was lower than expected. No statistically significant differences were found between the results of individual and group interventions.

**Trial registration number:**

ISRCTN32323770

## Background

Tobacco smoking is the leading cause of preventable death in developed countries. In Spain, more than 15% of all deaths are linked to smoking [1]. Reducing the use of tobacco is currently the most important public health measure that developed countries can implement. Because of their close contact with the public, primary health care workers can play a key role in efforts to reduce smoking.

From 1 to 3% of smokers quit smoking in six months after brief counseling by a health professional, while another 2% to 3% quit smoking with no help at all. Thus, counseling is a moderately effective public health intervention with a potentially large population impact [2]. Better results can be obtained with longer interventions [2]. Similarly, treatment with nicotine derivatives or with certain antidepressants (bupropion or nortriptyline) is effective [3,4] and doubles the chances of quitting smoking when used in conjunction with non-pharmacologic methods [5]. On smoker follow-up, abstinence rates as high as 20% are seen [6]. Cognitive behavior therapies [[7] and motivational interview techniques have also proved effective in getting people to quit smoking [8]. The combined use of all these smoking cessation methods, known as multi-component or intensive intervention [9], can lead to abstinence rates as high as 30% a year [10].

So far, no one has been able to demonstrate whether intensive group intervention is more effective than individual intervention [7]. A clinical trial conducted in our setting for the treatment of alcoholism showed group therapy to be more effective than individual therapy [11]. As for smoking behavior, two studies in our country have compared both types of interventions. The first was a clinical trial in which higher smoking cessation rates were obtained after six months with the group intervention than with the individual intervention, but the observed differences were not statistically significant [12]. The second was a quasi-experimental "pre-post" study without a control group in a specialized smoking cessation unit in which group therapy yielded a smoking cessation rate of 39% at 12 months, or better than the rate obtained in any study with the individual intervention [13].

Primary health care workers have little time to spare. Further study is needed to determine if group intervention is more effective than individual intervention because, if it turns out to be, implementing it as the preferred intervention for helping people quit smoking in health centers could save health care workers a considerable amount of time.

This study has two objectives. Its primary objective is to compare the effectiveness of intensive group and individual interventions for smoking cessation in primary health care. Its secondary objective is to identify the variables associated with smoking cessation.

## Methods

### Design

Three-pronged, randomized clinical trial in a primary health care setting in Mallorca. We carried out the following: an intensive individual intervention (III) and an intensive group intervention (IGI) as experimental branches, and the standard minimal intervention (MI) as the control branch.

### Subjects

The study population was made up of smokers who attended health centres. We established the following inclusion criteria: individuals who smoked and who were in the preparatory phase of smoking cessation in accordance with Prochaska's and Di Clemente's transtheoretical model of health behaviour change [14]. A prepared individual was defined as one who had expressed the wish to quit smoking and who felt ready to set a deadline for doing so not more than one month into the future. Individuals less than 18 years or with terminal illness or certain mental health conditions (dementia and schizophrenia) were excluded.

We expected a smoking cessation rate at 12 months of 5% in the MI, 15% in the III and 25% in the IGI. The sample size needed to detect a 10% difference in the main outcome measure between intervention groups with 5% precision in both directions, 80% power and a 95% confidence level was 199 subjects per intervention group, or 597 in all.

### Intervention

Three were conducted: III, IGI and MI. In all three, pharmacological treatment with nicotine derivatives or bupropion was offered as an option at the physician's discretion. Both the III and the IGI consisted of six visits during which the following were provided: counseling, psychological support and standard follow-up. Counseling and psychological support were based on motivational interview techniques [15] that sought to: (a) reinforce in the smoker the motivation to quit smoking before D day (the day fixed for quitting by the smoker) and (b) prevent relapses after smoking cessation. Intensive interventions followed clinical guidelines developed in the Balearic Islands [16,17]. Physicians and nurses in the III and IGI received identical training on how to implement intensive interventions, whereas health workers in the MI received only the basic training that had been offered previously to all primary health care workers on how to diagnose smoking addiction and provide brief counseling. In all three groups the intervention was carried out by the "microteam," composed of one physician and one nurse. These workers distributed the visits among themselves as they saw fit; all they were instructed to do was to conduct some of the visits together.

### Allocation method

An allocation concealment method based on the use of sequentially-numbered, opaque, sealed envelopes was used. All 40 health centers that existed in Mallorca at the time were invited to participate in the study, and 10 agreed. A block of 60 envelopes (20 for III, 20 for IGI and 20 for MI) was prepared in the central research unit for each participating health centre and subsequently sent out.

In each health centre, all physicians and nurses could recruit subjects, but only one "microteam" performed the III, one the IGI and one the MI. Smokers who fulfilled the inclusion and exclusion criteria were invited to participate in the study. If they consented, they were referred to the corresponding physician or nurse, who had them sign the informed consent form. After signing the form, patients picked an envelope at the admissions desk for random allocation to one of the intervention arms. Next, a visit with the doctor or nurse in the intervention arm to which the patient was allocated was scheduled, and the intervention was begun.

Once the intervention had ended, follow-up visits were scheduled at one month, 2 months, 3 months, 6 months, 9 months and 12 months. If a patient missed a follow-up visit, telephone follow-up was attempted. Case recruitment began in March 2005 and finished in June 2006. Case follow-up started in March 2005 and ended in August 2007.

### Main outcome measure

Continued abstinence at 12 months confirmed through CO. Secondary outcome measures: Self-reported continued abstinence at 12 months; point abstinence at 12 months confirmed by CO-oximetry (CO) and self-reported point abstinence at 12 months.

### Other variables

At baseline, the following information was obtained: (a) socio-demographic: age, sex, occupation and educational level; (b) lifestyle-related: use of alcohol and other drugs, practice of regular physical activity, fruit and vegetable intake; (c) health-related: history of arterial hypertension, diabetes, hyperlipidemia, obesity, asthma, COPD, ischemic heart disease, stroke, peripheral blood vessel disease, cancer and mental illness (type and use of psychotropic agents); (d) tobacco-related: main reason for wanting to quit smoking, number of cigarettes smoked daily, age at which smoking began, level of dependency as per Fagerström's test, smoking among individuals closest to the patient, number of past attempts to quit smoking, number of days of abstinence, strategy followed during past attempts (with or without professional help and with or without drugs) and reasons for relapse (weight gain, anxiety, insomnia, "smoking does no harm," etc.): (e) intervention-related: preferences in connection with the type of intervention they would have chosen had they been able to choose (individual, group, or either), and the strength of the patient's belief that he/she could quit smoking (on a scale from 0 to 10). During the intervention, the following information was obtained at each visit: the person in charge of conducting it (physician, nurse or both), its length, the number of cigarettes smoked, CO results, whether the participant was taking anti-smoking drugs (nicotine derivatives or bupropion) or not, and the strength of the patient's belief that he/she could quit smoking. Participants were classified as being on nicotine derivatives or bupropion if they were on these drugs during any of the intervention visits. At each follow-up visit, the number of cigarettes smoked and CO values were obtained.

### Statistical analysis

We used the chi squared test and the Anova and Kruskal-Wallis tests to ascertain whether randomization had resulted in three comparable groups at baseline, and to make comparisons across the three interventions. We used the Kolmogoroff-Smirnoff test to check the continuous variables for normal distribution.

For the main objective, the analysis was based on intention to treat. Cases lost during the interventions or lost to follow-up were treated as if they were still smokers at 12 months. The study statistician was blinded to the intervention allocation of the participants. Another member of the research team repeated the analysis to assess whether blinding was successful. Continued and point abstinence rates, confirmed by CO and self-report, were estimated. Relative risks were calculated for each outcome measure, along with the reduction in absolute risk and the number of individuals needed to treat (NNT) to obtain a single case of smoking cessation. All these measures are presented, along with their 95% confidence intervals.

To pursue the secondary objective, a bivariate analysis was performed to determine what variables were associated with smoking cessation. The chi squared test for qualitative variables and the Anova and Kruskal-Wallis tests for quantitative variables were used. Multivariate logistic regression was performed to determine if any variables other than group allocation were also associated with smoking cessation. Variables that yielded a significance level of < 0.25 in the bivariate analysis were selected by means of a backward LR. We evaluated at each step the potential confounding effect of the variables eliminated along the way. Multivariate analysis was repeated with forced entry of anti-smoking drugs into the model, and no changes in the final model beta coefficients were observed.

Statistical software SPSS 11.5 for Windows was used.

The study was performed with the approval of the Balearic Islands Ethics Committee.

## Results

We recruited 287 smokers: 81 in the III, 111 in the IGI, and 95 in the MI. Participants showed similar characteristics across all groups, both socio-demographically and in lifestyle, clinical history and smoking behavior (Additional file 1). Statistically significant differences were noted only with respect to their preferred interventions and COPD prevalence. The three interventions differed significantly (Additional file 2) in total number of visits; overall length (in minutes), which was six times greater in the IGI than in the III or MI; the health worker primarily responsible for conducting it, who was the physician in the III; the nurse, alone or with a physician, in the IGI; and the physician or nurse in the MI; and finally, the use of drug therapy, which was low in all cases, although it was highest in the MI, followed by the III, and lowest in the IGI. No adverse effects were observed.

**Figure 1:**
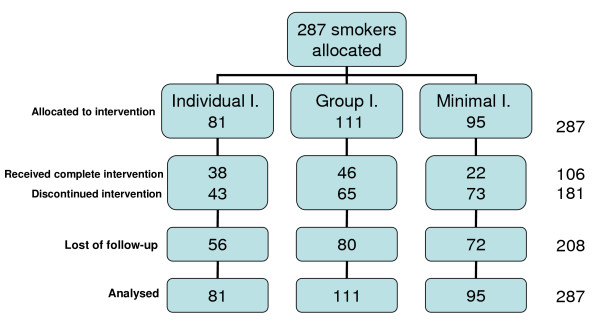
**Case flow**.

The flow of participants during the study is shown in Figure 1. Less than half of those who participated in the III and in the IGI completed the six intervention visits. Follow-up at 12 months was completed in 25 (31%) of cases in the III, 31 (28%) in the IGI and 23 (24%) in the MI. Only 10 participants in the III, 11 in the IGI and 3 in the MI showed up at all follow-up visits (or could be contacted by phone). CO was performed in 7 of the patients in the III, 6 in the IGI and 1 in the MI.

The results are displayed in Additional file 3, which shows that higher abstinence rates at 12 months were attained with the III than with the IGI and MI for each outcome measure, although III's superiority over the MI was only statistically significant in the case of self-reported continued abstinence and point abstinence confirmed by CO.

No differences were observed between the IGI and the III for continued abstinence confirmed by CO [RR 0.7 (0.2-2.2)] or for any of the other secondary outcome measures: self-reported continued abstinence [RR 0.8 (0.3-2.2)]; point abstinence confirmed by CO [RR 0.5 (0.2-1.1)], and self-reported point abstinence [RR 0.8 (0.4-1.8)].

Since self-reported point abstinence at 12 months was the outcome measure for which we had the greatest number of observations, we used it to determine if other factors were also associated with smoking cessation in the study population. For this outcome measure, the variables associated with smoking cessation in the bivariate analysis are shown in Additional file 4. Such variables were included in the multivariate analysis, along with sex, educational level and type of intervention. The final model chosen included, in addition to the type of intervention, the overall length of the visits and the number of past attempts to quit smoking. Only overall visit length showed a statistically significant association with smoking cessation (Additional file 5).

## Discussion

The results we obtained with intensive individual and group interventions were worse than expected. We wish to highlight two possible reasons for this: the lack of a consistent definition for individuals in the preparatory phase before smoking cessation, and the scarce use of drug therapy by health professionals.

Some individuals who were presumably in the preparatory phase dropped out of the interventions after the first visit or even before. This shows hat their level of "preparedness" was low. The strategy employed to ascertain preparedness status (asking individuals if they felt prepared and able to fix a date to quit smoking) may have been a poor one; it may have been better to assess the smoker's state of preparedness in greater depth through an open interview. Nonetheless, the different stages of the process of change are known to fluctuate [18]. In fact, authors such as Rollnick et al. [15] have suggested that preparedness should be viewed as a continuum along the process of smoking cessation instead of a well-defined phase. Thus, we may have incurred in selection bias by having used too simple a method to identify individuals who were prepared to quit smoking, whom we felt would be the ones to benefit the most from an intensive intervention.

An unequal number of subjects in each trial arm, together with differences among groups in subjects treatment preferences, could be another source of selection bias. The trial arms were not balanced because patients were unequally recruited among participating health centers, not because they were included only after being allocated to a trial arm that met their treatment expectations. Actually, the number of subjects who never attended an intervention visit after allocation was 4 in III, 1 in IGI and 2 in MI. The percentage of subjects who were allocated to an intervention in line with their expectations may have been due to chance.

Drugs (nicotine derivatives or bupropion) were used little during both individual and group interventions. Primary health care physicians' and nurses' perceptions of the effectiveness and safety of such treatments may be worth exploring, since according to Vogt et al., they may influence their prescription [19]. Perhaps not enough emphasis was placed on the effectiveness and benefits of drug therapy during health workers' training. It would have been a good idea, for instance, to provide them with illustrative experiences such as that of New York, where drugs were dispensed free of charge and abstinence rates greater than 20% were attained at one year [20]. On the other hand, the definition used for "being on drug therapy" - having been under treatment during one of the intervention visits, whether continuously or not - may explain why none of the individuals being treated with nicotine derivatives quit smoking.

We do not feel the poor results obtained are part of the described trend towards decreased effectiveness of anti-smoking treatments because non-quitters are the more recalcitrant individuals who never succeed in shaking the addiction [21]. In our setting, the last twenty years have seen a drop in smoking prevalence, especially in males [22], although the prevalence rate continues to be high, particularly among the lower social classes [23]. In the Balearic Islands in particular, 33.7% of the men and 20.3% of the women report being daily smokers [24].

Individual intervention yielded somewhat better results than group intervention, which is six times as long. This is precisely the disadvantage of group interventions, since a large percentage of patients drop out in the course of the sessions. As shown in this study, however, the time invested by health workers was the only factor associated with smoking cessation. Thus, adherence to treatment is crucial, as already demonstrated by other studies in which a dose-response relationship was found between the number of visits and a successful outcome [25]. Studying why participants drop out of sessions would be useful in trying to prevent such losses and in learning to appropriately choose candidates for intensive interventions.

Based on our findings, no specific type of intervention can be recommended to help smokers who attend health centers to quit smoking. It is probably best for health centers to provide group as well as individual interventions, and minimal interventions in addition to intensive ones. On the one hand, repeated minimal intervention is very cost-effective [26]; on the other, group intervention requires more available resources, such as nursing staff. Individual intervention could be reserved for smokers who demand individualized care [27], although it is worth remembering that smokers can quit smoking on their own and that health care workers as well as public health officials should encourage them to do so and pass laws restricting the use of tobacco products in public spaces [28].

The study has two main limitations: non-attainment of the estimated sample size and losses to follow-up.

We were unable to attain the desired sample size because during the study more than 20% of participating doctors and nurses were transferred to other health centers. As a result, we had to identify new professionals to recruit smokers and train them in conducting smoking cessation interventions. Our study lacked sufficient power to detect statistically significant differences between the results of intensive individual and group interventions, but the findings suggest that we were wrong in our hypothesis that intensive group intervention for smoking cessation is more effective than intensive individual intervention.

When we compared self-reported point abstinence rates with continued abstinence rates confirmed with CO, we observed better results (12% for III, 10% for IGI and 6% for MI compared with 7% for III, 5% for IGI and 1% for MI). We consider self-reported point abstinence rates more realistic and similar to those obtained in previous studies. Although continuous abstinence is considered the gold standard outcome for smoking cessation [29], some studies have shown that smokers do not lie about their smoking status when asked about it directly [30].

Patient follow-up presented additional difficulties, both for patients who quit smoking and for those who did not. Participants in the first group did not understand why they had to continue visiting the health centre since they had quit smoking already; those in the second group felt embarrassed about not having been able to quit. Poor compliance with follow-up visits made us choose point self-reported smoking cessation as the outcome measure for exploring other variables potentially associated with having quit smoking.

## Conclusions

Intensive smoking cessation interventions in this study were less effective than expected. No statistically significant differences could be shown between the results of individual and group interventions. Further research is needed on the effectiveness of combination treatments to help individuals quit smoking in primary health care settings.

## Competing interests

The authors declare that they have no competing interests.

## Authors' contributions

MR liderated the design and development of the study, helped in the statistical analysis and draft the manuscript. JR performed the statistical analysis and critically reviewed the draft. TE, IS, AF and RD coordinated the development of the study in their health centres, recruited cases, performed interventions and critically reviewed the draft. MJG and MS recruited cases, performed interventions and critically reviewed the draft. All authors read and approved the final manuscript.

## Pre-publication history

The pre-publication history for this paper can be accessed here:

http://www.biomedcentral.com/1471-2458/10/89/prepub

## Supplementary Material

Additional file 1 **Group comparability before the intervention**. The data provided represent the characteristics of the participants in each branch before the intervention. Click here for file

Additional file 2 **Group comparability after the intervention**. The data provided represent the characteristics of the intervention received in each branch. Click here for file

Additional file 3 **Intervention effectiveness**. The data provided represent the outcome measures for the main objective of the study. Click here for file

Additional file 4 **Variables associated with smoking cessation in the bivariate analysis**. The data provided represent the bivariate analysis for the secondary objective. Click here for file

Additional file 5 **Variables associated with smoking cessation in the multivariate analysis**. The data provided represent the multivariat analysis for the secondary objective. Click here for file
