# Supplementary material for: Effectiveness of intensive group and individual interventions for smoking cessation in primary health care settings: a randomized trial
Source: BMC Public Health. 2010 Feb 23;10:89. doi: 10.1186/1471-2458-10-89 (PMC2836298; doi:10.1186/1471-2458-10-89)
Supplement: Additional file 1 — Group comparability before the intervention. The data provided represent the characteristics of the participants in each branch before the intervention. [file 1471-2458-10-89-S1.RTF]

Additional file 1. Group comparability before the intervention
Variables		Individual I. N=81  n (%)	Group I.
 N=111  n (%)	Minimal I.
N=95  n (%)	
Sex		Men	35 (43.2)	47 (42.7)	50 (52.6)	
                                	Women	46 (56.8)	63 (57.3)	45 (47.4)	
                              				
Educational level	Elementary	39 (50.6)	64 (61.0)	40 (44.4)	
                                	High school	29 (37.7)	33 (31.4)	42 (46.7)	
                                	Higher	9 (11.7)	 8 (7.6)	8 (8.9)	
				
Reason for quitting smoking 	Health	38 (52.8)	60 (62.5)	53 (60.2)	
                      				
Smoker present in the immediate surroundings	31 (39.2)	52 (49.1)	42 (46.2)	
				
Has tried to quit smoking in the past	69 (85.2)	82 (74.5)	77 (81.1)	
				
Method used	Alone	65 (94.2)	71 (89.9)	65 (86.7)	
                               	With professional help	4 (5.8)	8 (10.1)	10 (13.3)	
                               				
Reason for failure	Anxiety	38 (55.9)	34 (43.0)	34 (45.9)	
                               	“Just one does no harm”	23 (33.8)	25 (31.6)	27 (36.5)	
                               				
Fagerstrom test	Low dependency	17 (21.8)	15 (13.8)	22 (24.2)	
                               	Moderate dependency	35 (44.9)	48 (44.0)	42 (46.2)	
                               	Strong dependency	26 (33.3)	 46 (42.2)	27 (29.7)	
				
Type of intervention preferred by the subject				
                                  Group	10 (13.3)	43 (43.4)	7 (7.5)	
                                  Individual	27 (36.0)	17 (17.2)	46 (49.5)	
                                  Does not care	38 (50.7)	39 (39.4)	40 (43.0)	
				
Eats fruits and vegetables daily	53 (67.1)	81 (79.4)	64 (68.1)	
Walks daily	 47 (58.8)	59 (58.4)	47 (52.2)	
Practices sports	17 (22.7)	23 (21.7)	16 (17.4)	
Drinks alcoholic beverages regularly	 26 (32.5)	22 (21.2)	25 (26.9)	
Uses marijuana	8 (11.0)	8 (8.0)	5 (6.2)	
Uses cocaine	1 (1.4)	0	1 (1.3)	
Hypertension	11 (15.9)	11 (12.6)	11 (12.6)	
Diabetes	6 (8.6)	4 (4.6)	6 (7.0)	
Dyslipidemia	16 (22.9)	19 (21.6)	16 (18.6)	
Obesity	10 (16.1)	12 (14.0)	12 (14.5)	
Chronic Obstructive Pulmonary Disease (EPOC)	10 (14.7)	4 (4.7)	3 (3.6)	
Ischaemic heart disease	3 (4.5)	3 (3.5)	1 (1.2)	
Stroke	0	0	1 (1.2)	
Peripheral vascular disease	0	0	2 (2.4)	
Cancer	2 (3.0)	3 (3.5)	3 (3.6)	
Asthma	4 (6.3)	6 (6.9)	5 (5.8)	
Treatment with psychoactive drugs	15 (20.0)	25 (27.5)	14 (16.1)	
Mental health problems	22,4	30,5	16,1	
                              Depression	4 (4.9)	10 (9.0)	7 (7.4)	
                              Mixed anxiety-depression disorder 	1 (1.2)	4 (3.6)	2 (2.1)	
                              Anxiety	6 (7.4)	11 (9.9)	3 (3.2)	
				
Mean age  	44.5 10.9	45.0  10.9	43.9  9.9	
Median no. of cigarettes smoked daily 	20 (15.0-30.0)	20 (18.0-30.0)	20 (17.8-30.0)	
Median age when began smoking  	16 (14.0-18.0)	16 (13.0-18.0)	16 (15.0-18.2)	
Median no. of quitting attempts 	1 (1.0-3.0)	2 (0-3.0)	2 (1.0-3.0)	
Median no. of days of abstinence 	30 (2.5-196.0)	30 (0-180.0)	60 (3.0-270.0)	
