# Supplementary material for: Effectiveness of intensive group and individual interventions for smoking cessation in primary health care settings: a randomized trial
Source: BMC Public Health. 2010 Feb 23;10:89. doi: 10.1186/1471-2458-10-89 (PMC2836298; doi:10.1186/1471-2458-10-89)
Supplement: Additional file 2 — Group comparability after the intervention. The data provided represent the characteristics of the intervention received in each branch. [file 1471-2458-10-89-S2.RTF]

Additional file 2. Group comparability after the intervention
Variables	Individual I.
n (%)	Group I.
n (%)	Minimal I.
n (%)	
Professional conducting the visit	Physician	56 (83.6)	1 (1.1)	47 (56.6)	
	Nurse	11 (16.4)	44 (50.6)	30 (36.1)	
	Both	0	42 (48.3)	6 (7.2)	
				
Drug therapy                                 Yes	21 (25.9)	22 (19.8)	35 (36.8)	
				
Bupropion                                      Yes	17 (21.0)	14 (12.6)	26 (27.4)	
				
Nicotinic agents                             Yes	4 (4.9)	9 (8.1)	10 (10.5)	
				
Total number of visits	5 (2-6)	5 (2-6)	3 (1-5)	
				
Total length of visits (in minutes)	50 (32.5 – 80.0)	315 (120.0-470.0)	40 (20.0-77.0)	
