# Supplementary material for: Effectiveness of intensive group and individual interventions for smoking cessation in primary health care settings: a randomized trial
Source: BMC Public Health. 2010 Feb 23;10:89. doi: 10.1186/1471-2458-10-89 (PMC2836298; doi:10.1186/1471-2458-10-89)
Supplement: Additional file 3 — Intervention effectiveness. The data provided represent the outcome measures for the main objective of the study. [file 1471-2458-10-89-S3.RTF]

Additional file 3. Intervention effectiveness

	No. and % of abstinent subjects	RR	ARR	NNT	
	III
(N=81)	IGI
(N=111)	MI
(N=95)	III	IGI	III	IGI	III	IGI	
Continued abstinence:	
Confirmed by CO	6 (7.4)	6 (5.4)	1 (1.0)	7.04 (0.9-7.2)	5.1 (0.6-41.9)	6.4 (0.3-12.4)	4.4 (-0.3-9.0)	16 (8.0-333.0)	23 (-333.0-11.0)	
Self-reported	7 (8.6)	8 (7.2)	1 (1.0)	8.2 (1.03-65.3)	6.8 (0.9-53.8)	7.6 (1.1-14.0)	6.2 (0.9-11.4)	13 (7.0-91.0)	16 (9.0-111.0)	
Point abstinence:	
Confirmed by CO	11 (13.6)	7 (6.3)	3 (3.2)	4,3 (1.2-14.9)	2 (0.5-7.5)	10,4 (2.2-18.7)	3.1 (-2.6-8.9)	10 (5.0-45.0)	32 (-38.0-11.0)	
Self-reported	10 (12.3)	11 (9.9)	6 (6.3)	1.9 (0.7-5.1)	1.6 (0.6-4.1)	6 (-2.6-14.7)	3.6 (-3.8-11.0)	17 (-38.0-7.0)	28 (-26.0-9.0)	
RR: Relative risk; ARR: Absolute risk reduction; NNT: Number needed to treat in order to observe a single case of smoking cessation; III: Intensive individual intervention; IGI: Intensive group intervention; MI: Minimal intervention; CO: CO-oximetry.
