# Supplementary material for: Effectiveness of intensive group and individual interventions for smoking cessation in primary health care settings: a randomized trial
Source: BMC Public Health. 2010 Feb 23;10:89. doi: 10.1186/1471-2458-10-89 (PMC2836298; doi:10.1186/1471-2458-10-89)
Supplement: Additional file 4 — Variables associated with smoking cessation in the bivariate analysis. The data provided represent the bivariate analysis for the secondary objective. [file 1471-2458-10-89-S4.RTF]

Additional file 4. Variables associated with smoking cessation in the bivariate analysis
		Non-smokers
n (%)	p	
Dichotomic variables			
Sex       Men	13 (9.8)	0.827	
             Women	 14 (9.1)		
Has tried to quit smoking in the past	 25 (11.0)	0.080	
Smoker present in the immediate surroundings	10 (8.0)	0.364	
Visits attended 	 21 (13.6)	0.008	
Treatment with nicotinic agents	0 (0.0)	0.107	
Teatment with bupropion	 9 (15.8)	0.065	
Walks daily	 16 (10.5)	0.583	
Practices sports	6 (10.7)	0.817	
Eats fruits and vegetables daily	 19 (9.6)	0.843	
History of prior illness	   10 (8.6)	0.707	
Mental health problems	 5 (8.2)	0.699	
			
Dummy variables			
Intervention	Intensive individual	 10 (12.3)	0.383	
	Intensive group	 11 (9.9)		
	Minimal	 6 (6.3)		
				
Educational level	Elementary	16 (11.2)	0.455	
	High School	 7 (6.7)		
	Higher	3 (12.0)		
				
Fagerstrom test	Low dependency	1 (1.9)	0.096	
                               	Moderate dependency	15 (12.0)		
                               	Strong dependency	10 (10.1)		
				
Professional conducting the visit	Physician	12 (11.5)	0.236	
	Nurse	4 (4.7)		
	Both	5 (10.4)		
				
Quantitative variables*		Non-smokers	Smokers		
Age		44 (11.4)	44.6 (10.5)	0.817	
No. of cigarettes smoked daily		20 (15.0-25.0)	20 (17.0-30.0)	0.316	
Total no. of visits		6 (5.0-6.0)	4 (2.0-6.0)	0.000	
Total length of visits (in minutes)		112 (57.0-600.0)	70 (25.0-163.7)	0.007	
No. of visits conducted by the two professionals 		0 (0.0-3.0)	0 (0.0-1.0)	0.109	
Degree of confidence		8 (6.5-9.2)	7,4 (5.8-8.8)	0.087	
*Mean (with standard deviation) or median (with percentiles 25-75)
