# Supplementary material for: Effectiveness of intensive group and individual interventions for smoking cessation in primary health care settings: a randomized trial
Source: BMC Public Health. 2010 Feb 23;10:89. doi: 10.1186/1471-2458-10-89 (PMC2836298; doi:10.1186/1471-2458-10-89)
Supplement: Additional file 5 — Variables associated with smoking cessation in the multivariate analysis. The data provided represent the multivariat analysis for the secondary objective. [file 1471-2458-10-89-S5.RTF]

	â	p	Expâ	95% CI	
Intervention		0.041			
             Individual I.	0.692	0.204	1.997	0.687-5.807	
             Group I.	-1.504	0.109	0.222	0.035-1.399	
Has tried to quit in the past	1.324	0.087	3.759	0.824-17.149	
Total length of visits	0.006	0.001	1.006	1.003-1.010	
Additional file 5. Variables associated with smoking cessation in the multivariate analysis
